# Mini-open compared with the trans-tubular approach in patients with spinal metastases underwent decompression surgery---a retrospective cohort study

**DOI:** 10.1186/s12885-023-11730-4

**Published:** 2023-12-13

**Authors:** Yunpeng Cui, Chuan Mi, Bing Wang, Yuanxing Pan, Yunfei Lin, Xuedong Shi

**Affiliations:** Department of Orthopaedics, Peking University First Hospital, Beijing, China

**Keywords:** Spinal metastases, Mini-open, Trans-tubular, Minimally invasive spine Surgery

## Abstract

**Background:**

This study aimed to evaluate the perioperative safety and efficacy of the Mini-open and trans-tubular approach in patients with spinal metastases who underwent decompression surgery.

**Methods:**

37 consecutive patients with spinal metastases who underwent decompression surgery through a Mini-open or trans-tubular approach were retrospectively reviewed between June 2017 and June 2022. Thirty-four patients were included in this study. 19 underwent decompression surgery through the Mini-open approach, and 15 underwent the Trans-tubular approach. T-test and chi-square test were used to evaluate the difference between baseline data and primary and secondary outcomes.

**Results:**

Baseline characteristics did not differ significantly between Trans-tubular and Mini-open groups except for the Ambulatory status (*P* < 0.001). There was no significant difference in blood loss between the two groups (*P* = 0.061). Operative time, intraoperative blood transfusion, intraoperative complication (dural tear), and postoperative hospitalization were comparable in the two groups (*P* > 0.05). The trans-tubular group had significantly less amount of postoperative drainage (133.5 ± 30.9 ml vs. 364.5 ± 64.2 ml, *p* = 0.003), and the time of drainage (3.1 ± 0.2 days vs. 4.6 ± 0.5 days, *p* = 0.019) compared with Mini-open group (*P* < 0.05). Sub-group analysis showed that for patients with hypo-vascular tumors, the Trans-tubular group had significantly less blood loss than the Mini-open group (951.1 ± 171.7 ml vs. 1599.1 ± 105.7 ml, *P* = 0.026).

**Conclusions:**

Decompression through Mini-open or Trans-tubular was safe and effective for patients with spinal metastases. The trans-tubular approach might be more suitable for patients with hypo-vascular tumors.

## Background

The spine was one of the most common sites of bone metastasis. The number of patients with spinal metastasis remarkably increased due to the large number of patients and survival [1]. Bone protectants such as denosumab and zoledronic acid had significantly reduced the incidence of skeletal-related events [2, 3]. However, surgery was still the first choice for patients with pathological fractures, spinal cord, or nerve root compression to improve patients’ symptoms and quality of life rapidly.

For most patients with spinal metastases, the surgery aimed to improve the quality of life rather than radically remove local lesions. Conventional surgery was effective for symptomatic spinal metastases accompanied by higher postoperative complications [4, 5]. Rapid rehabilitation and high quality of life were essential for subsequent treatment, such as radiation, chemotherapy, or targeted drugs for patients with advanced cancer.

More spinal tumor surgeons performed minimally invasive surgical methods for patients with spinal metastases. The core purpose of minimally invasive treatment was to reduce muscle tissue peeling. Pedicle screws were percutaneously placed under fluoroscopy or freehand pedicle screw fixation. Decompression was mainly performed using small incision [6–8] and trans-tubular [9–11]. These two minimally invasive surgical methods had achieved positive therapeutic results in clinical practice. However, there was no literature to compare these two minimally invasive methods. In clinical work, it needed to be clarified how to choose between these two methods. The present study compared the effectiveness and safety of these minimally invasive surgeries (mini-open and trans-tubular). The results would guide surgeons in selecting the appropriate method.

## Methods

### Study design and selection criteria

This was a single-centered retrospective cohort study of patients with spinal metastases who underwent decompression surgery through a mini-open or trans-tubular approach in our department from June 2017 to June 2022. Patients were assigned to two groups according to the decompression approach: The mini-open approach and the Trans-tubular approach.

Exclusion criteria:

1. Patients with recurrent tumors;
2. Patients who simultaneously underwent conventional decompression surgery at the same or different segment;

### Data collection

We reviewed the medical records to assess general, operational, laboratory, and functional data among the clinical variables. General data included age, gender, ASA (American Society of Anesthesiologists physical status classification system), tumor pathology, clinical manifestation, and Tokuhashi score. Laboratory data included preoperative and postoperative HGB, HCT, and ALB. Operation-related data included the blood loss, the location, and number of decompression segments, decompression approach, operation time, blood transfusion on an operative day, drainage amount, complications, and postoperative hospitalization. According to AIS, functional status data included the Visual Analogue Scale (VAS), Karnofsky score, ambulatory status, and neurological function. Metastasis from renal, liver, and thyroid tumors was assigned to the hyper-vascular tumor [12].

### Surgical procedure

An experienced spinal tumor surgeon under general anesthesia performed all surgeries. Pedicle screws were percutaneously placed under fluoroscopy or freehand pedicle screw fixation [13]. Circum-spinal decompression was achieved through the trans-tubular approach and the Mini-open approach. The trans-tubular approach was performed through a unilateral paraspinal muscle space [10]. The operation was performed through a unilateral paravertebral muscular space approach with an expandable tubular retractor and a cold light source. This approach could preserve the integrity of the bony structures, including the spinous process, the contralateral vertebral plate, and the lesser articulations, and protect the attachment of the paravertebral musculature. Before placing the tubular retractor, the pedicles of the diseased vertebrae were precisely localized with a K-wire. Depending on the degree of obesity, a longitudinal incision was made 2 cm or more lateral to the skin projection of the pedicle. After the incision of the deep fascia, the paravertebral muscles were bluntly separated, and a tubular retractor was placed. Appropriate muscle stripping revealed the diseased vertebrae’s transverse processes and small joints. If the transverse processes, small joints, and pedicles were not involved with the tumor, those were removed in pieces to expose the tumor. Spatula and osteotome were used to remove the tumor in the vertebral body carefully, and the tumor protruded into the vertebral canal behind the vertebral body by piecemeal excision. If the small joints and the transverse processes were involved, the transverse processes, small joints, and part of the pedicles that the tumor had invaded could be resected directly, the nerve roots should be exposed and protected, and the lateral side of the dural sac should be exposed. The mini-open approach was only through a small skin incision at the decompression segment [8]. The mini-open approach was performed via the median posterior approach only at the decompression segment. The deep fascia was cut longitudinal along the incision. Sacrospinalis muscles were stripped from the bone surface to expose the posterior structures. The posterior structures and pedicles of the vertebra were removed by piecemeal excision to expose the dural sac and tumor. Spatula and osteotome were used to carefully remove the pedicle tumor, the vertebral body, and the tumor protruding into the vertebral canal behind the vertebral body by piecemeal excision. The nerve root should be exposed and protected.

### Outcome measurement

The primary outcome was total blood loss. The Gross equation calculated the total blood loss [14].

Gross equation: total perioperative blood loss = theoretical total blood loss + allogeneic blood transfusion. Patients in this study did not use autologous blood transfusions during and after surgery.

Theoretical total blood loss = estimated blood volume × 2 × (preoperative Hct-postoperative Hct) / (preoperative Hct + postoperative Hct). Postoperative Hct was examined on the first morning after the operation.

Patient’s estimated blood volume = k1×height (m)³+k2×weight (kg) + k3 [15].

Male patients k1 = 0.3669, k2 = 0.03219, k3 = 0.6041; Female patients k1 = 0.3561, k2 = 0.03308, k3 = 0.1833.

Other perioperative data were set as secondary outcomes.

- the amount of intraoperative blood transfusion.
- the amount and time of drainage.
- the length of postoperative hospitalization.
- intraoperative complication (dural tear).
- the improvement of functional status.

The intraoperative indication for transfusion in our hospital was HGB less than 80 g/L in general or 90 g/L for patients with coronary heart disease.

### Statistical analysis

Continuous variables were presented as mean ± SE. Categorical variables were expressed as numbers. Independent sample t-test was used to detect the difference among continuous variables. The differences among the categorical variables were analyzed using the chi-square test or Fisher’s Exact Test (n < 40). All tests were on 2 sides. A p-value < 0.05 was considered statistically significant. Patients with missing data related to primary outcomes were excluded from the study. Delete cases with missing data of other values during the statistical process. Delete cases with missing values during the statistical process. Data were analyzed with SPSS 25.0 statistical software (IBM Corporation, Armonk, NY.).

## Results

### Patient enrollment

**Fig. 1:**
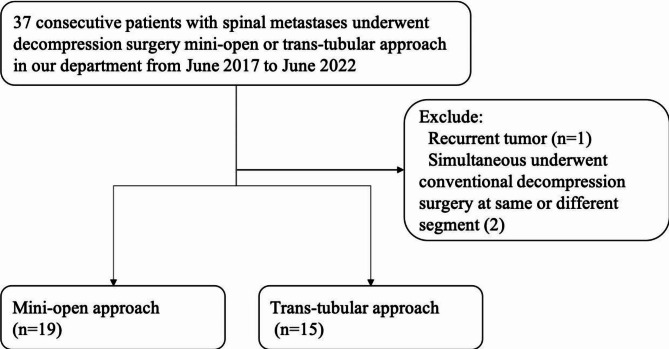
The flowchart of patient inclusion

Thirty-seven consecutive patients were enrolled as our initial cases. We excluded those patients with recurrent tumors (1 case) and simultaneous conventional decompression surgery (2 cases). At last, a total of 34 patients were included in this study. Nineteen patients underwent decompression surgery through the Mini-open approach, and 15 patients underwent decompression surgery through the Trans-tubular approach. The flow of patient enrolment is shown in Fig. 1.

### Patient’s baseline data

The study included 34 patients with a mean age of 66.0 ± 1.9 years. There were 25 males and nine females. The detailed baseline data were shown in Tables 1 and 2, and Table 3. Except for Ambulatory status, there were no significant differences between the Trans-tubular and Mini-open groups’ demographic, preoperative tumor-related data, functional status, and laboratory data. The trans-tubular group had more patients who could walk (80% vs. 52.6%, *P*<0.001) than the Mini-open group.

**Table 1:** Baseline data

Characteristic	MO (n = 19)	TA (n = 15)	*P*
Age	66.4 ± 2.2	65.4 ± 3.3	0.792
Gender			
Female	4	5	0.462
Male	15	10	
ASA score			
II	8	10	0.185
III	11	5	
Tumor pathology			
Renal	2	4	0.331
Lung	5	4	
Prostate	8	2	
Multiple myeloma	1	3	
Uroepithelium	1	1	
Esophageal	0	1	
Colon	1	0	
Breast	1	0	
Blood supply			
Hypervascular (renal)	2	4	0.370
Non-hyper vascular (non-renal)	17	11	
Clinical manifestation			
Spinal nerve root symptom	15	14	0.355
Spinal cord symptom	4	1	
Tokuhashi score	7.7 ± 0.5	7.5 ± 0.6	0.784

**Table 2:** Laboratory data

Characteristic	MO (n = 19)	TA (n = 15)	*P*
**Pre-operation**			
HGB (g/L)	120.5 ± 3.5	120.1 ± 5.6	0.951
Hct (%)	35.9 ± 1.1	36.2 ± 1.8	0.907
Alb (g/L)	39.1 ± 0.9	37.9 ± 1.2	0.447
**Decline (Postoperative day 1)**			
HGB decline (g/L)	20.8 ± 2.0	17.4 ± 4.3	0.481
Hct decline (%)	6.5 ± 0.6	5.6 ± 1.3	0.537
Alb decline (g/L)	7.8 ± 0.7	6.7 ± 1.1	0.401

HGB, haemoglobin; Hct, haematocrit; Alb, albumin

**Table 3:** Functional status

Characteristic	MO (n = 19)	TA (n = 15)	*P*
**Pre-operation**			
VAS	7.7 ± 0.2	7.9 ± 0.1	0.573
Karnofsky score	56.8 ± 1.7	58.7 ± 1.7	0.458
Ambulatory status			
Nonambulatory	9	3	**<0.001**
Ambulatory (assistant)	10	3	
Ambulatory	0	9	
AIS			
C	1	0	0.215
D	2	2	
E	1	5	
**Post-operation improvement**			
VAS	2.9 ± 0.2	2.9 ± 0.4	0.927
Karnofsky score	61.6 ± 1.4	64.8 ± 4.8	0.485
Ambulatory status			
Improve	14	5	**0.036**
Same	5	10	
Aggravate	0	0	
AIS			
Improve	1	5	0.242
Same	3	2	
Aggravate	0	0	

AIS, American Spinal Injury Association Impairment Scale; VAS, Visual Analogue Scale

### Primary outcome

The surgical outcomes were shown in Table 4. There were no significant differences in the incidence of preoperative arterial embolism, the location of the lesion, and the number of decompression segments between the Trans-tubular and Mini-open groups. Although it did not reach a statistically significant level, the Trans-tubular group had less blood loss than the Mini-open group (1192.3 ± 183.2 ml vs. 1571.9 ± 97.4 ml, *P* = 0.061). We also conducted a sub-group analysis to clarify the relevance of the tumor’s vascular supply to the blood loss of the two groups (Table 5). For patients with hyper-vascular tumor (renal), the Trans-tubular group had more blood loss than the Mini-open group (1855.6 ± 342.4 ml vs. 1340.8 ± 203.2 ml, *P* = 0.385). For patients with hypo-vascular tumors, the Trans-tubular group had significantly less blood loss than the Mini-open group (951.1 ± 171.7 ml vs. 1599.1 ± 105.7 ml, *P* = 0.026).

**Table 4:** Surgical data

Characteristic	MO (n = 19)	TA (n = 15)	*P*
Location of the lesion			
Lumbar spine	15	8	0.151
Thoracic spine	4	7	
Number of decompression segment			
1	15	14	0.355
2	4	1	
Preoperative embolism			
Yes	2	2	1.000
No	17	13	
Dural tear			
Yes	0	2	0.187
No	19	13	
Operative time (min)	278.7 ± 9.6	254.5 ± 16.6	0.194
Blood loss(ml): Gross equation	1571.9 ± 97.4	1192.3 ± 183.2	0.061
Intraoperative blood transfusion (ml)	673.7 ± 68.8	480.0 ± 97.2	0.104
Drainage: Amount (ml)	364.5 ± 64.2	133.5 ± 30.9	**0.003**
Drainage: Time (day)	4.6 ± 0.5	3.1 ± 0.2	**0.019**
Postoperative hospitalization (day)	10.3 ± 2.4	9.0 ± 0.9	0.640

Note: Two patients in TA group with dural injury were excluded when calculating the drainage volume

**Table 5:** Sub-analysis of surgical data

Characteristic	Hyper-vascular tumor (renal)			Hypo-vascular tumor (non-renal)		
	MO (n = 2)	TA (n = 4)	*P*	MO (n = 17)	TA (n = 11)	*P*
Operation time (min)	308.5 ± 5.5	315.8 ± 40.8	0.911	275.2 ± 10.4	232.3 ± 12.5	0.015
Blood loss(ml): Gross equation	1340.8 ± 203.2	1855.6 ± 342.4	0.385	1599.1 ± 105.7	951.1 ± 171.7	0.026
Intraoperative Blood transfusion (ml)	600.0 ± 200.0	700.0 ± 251.6	0.813	682.3 ± 74.9	400.0 ± 93.4	0.002

### Secondary outcomes

There were no significant differences in the operative time, intraoperative blood transfusion, intraoperative complication (dural tear), and postoperative hospitalization between the Trans-tubular and Mini-open groups (Table 4). The trans-tubular group had significantly less amount of postoperative drainage (133.5 ± 30.9 ml vs. 364.5 ± 64.2 ml, *p* = 0.003), and the time of drainage (3.1 ± 0.2 days vs. 4.6 ± 0.5 days, *p* = 0.019) compared with Mini-open group. For patients with hyper-vascular tumors (renal), the operation time and intraoperative blood transfusion were comparable between the two groups. For patients with hypo-vascular tumors, patients in the Trans-tubular group had significantly less operation time (232.3 ± 12.5 min vs. 275.2 ± 10.4 min, *P* = 0.015) and intraoperative blood transfusion (400.0 ± 93.4 ml vs. 682.3 ± 74.9 ml, *P* = 0.002) compared with patients in Mini-open group (Table 5). There were no significant differences in HGB, Hct, and Alb between the two groups on postoperative day 1. However, HGB, Hct, and Alb declined more in the Mino-open group. Except for Ambulatory status, there were no significant differences in the functional improvement of AIS, VAS, and Karnofsky scores between the two groups. The mini-open group had better improvement in Ambulatory status than the Trans-tubular group (73.7% vs. 33.3%, *P*<0.036).

## Discussion

More and more spinal tumor surgeons use minimally invasive methods to treat metastatic spinal tumors. Minimally invasive surgery can achieve the same therapeutic effect regarding safety, pain reduction, and neurological outcome compared to conventional surgery [16]. The present study comprehensively analyzes the two main minimally invasive surgery methods: mini-open and trans-tubular. This study showed that both mini-open and trans-tubular surgery were effective and safe for patients with spinal metastases.

The gross equation was used to calculate the perioperative blood loss. This study showed that the trans-tubular group had less blood loss, intraoperative allogeneic blood transfusion, and operation time than the mini-open group for non-hypervascular tumors. Conversely, the trans-tubular group had more blood loss, intraoperative allogeneic blood transfusion, and operation time than the mini-open group for patients with hypervascular tumors. Both mini-open and trans-tubular approaches were intratumorally resectioned. The hyper-vascular tumor had more bleeding at the tumor wound and needed to be compressed to stop bleeding. While pressing, avoiding the spinal cord, nerve root, and other vital structures was necessary. Due to the limited field of vision under the tubular, hemostasis would not be effectively performed before the exposure of important structures such as the spinal cord and nerve root. Compared with mini-open surgery, tubular surgery had stricter criteria on tumor type. For patients with hyper-vascular tumors, the mini-open technique should be given priority. However, the trans-tubular group had less drainage and faster postoperative recovery than the mini-open group, regardless of the tumor’s blood supply. Preoperative arterial embolism or other interventions may be performed to control the bleeding during operation for patients with hyper-vascular tumors [12].

In this study, the dural sac was more easily injured during the operation. The learning curve for utilizing an emerging technique must be considered when performing trans-tubular surgery. Silva PS et al. analyzed 150 patients with degenerative lumbar disease who underwent MI-TLIF. The most frequent complication was a dural tear (5.32%); the complication rates were 33% and 20.51% for 50% and 90% of learning milestones, respectively. They reported that 90% of the learning curve would be achieved around the 40th case [17]. Lin J et al. analyzed 62 patients with spinal metastases who underwent mini-open surgery. The operative time decreased gradually with the number of surgical cases increasing and stabilized after the 20th patient [13]. The complication rate will improve with the growth of this trans-tubular learning curve in the future.

The trans-tubular group had better preoperative ambulatory status than the mini-open group in this study. The poor ambulatory status was often caused by severe compression and poor compensatory ability of the spinal cord. Severe compression was often accompanied by severe deformation of the dural sac and the boundary between the dural sac and the tumor tissue was unclear. Like traditional surgery, mini-open surgery would remove part of the adjacent vertebral lamina to expose the normal dural sac. Then, the boundary of the dural sac could be confirmed. The visibility under the tubular was limited. It was difficult to effectively expose the typical structures of the upper and lower vertebrae and the normal boundary of the dural sac. In this study, the dural sac was more easily injured during the operation.

Lee et al. retrospective analysis of 131 patients with MSCC. The results showed that the local recurrence rate of patients in the en-bloc resection group did not exceed 20%, while the local recurrence rate of patients in the piece-meal resection group increased year by year with the prolongation of the follow-up time and could reach more than 60% at 4 years postoperatively. The difference in the local recurrence rate between the two groups was statistically different [18]. However, The complications of en-bloc resection were higher. They would affect subsequent adjuvant therapy [19].

Compared with the mini-open approach, the Trans-tubular approach had disadvantages in intraoperative visualization. On the other hand, the Trans-tubular approach did not achieve circumferential decompression of the dural sac due to the retention of the normal structure of the contralateral side. There was a higher risk of local recurrence and recompression of patients performing the Trans-tubular approach. Effective postoperative systemic therapy, as well as radiotherapy, were even more important for these patients. Radiotherapy was effective as a means to reduce the local recurrence of tumors [20]. The meta-study results showed a 1-year local recurrence rate of 10% after surgery combined with radiotherapy for MSCC. Surgery combined with radiotherapy was considered a standard treatment option for patients with MSCC. We recommend routine postoperative radiation therapy for patients performing piece-meal resection [21].

The mini-open approach was only through a small skin incision at the decompression segment. The pedicle screw was inserted through the paravertebral muscle space. The small incision was deficient in visualizing the tumor mass if a mass of tumor tissue formed in the posterior structures of the spine. This approach should better be used in patients with no mass of tumor tissue formed in the posterior structures of the spine. The trans-tubular approach was performed through a unilateral paraspinal muscle space. The application scenarios were more limited for this approach. First, the responsible lesion was only at a single segment, and the lesion was predominantly located unilaterally and can be decompressed by unilateral tumor resection. Second, effective medical systemic therapy or local radiotherapy can be implemented postoperatively. Bilateral or circumferential compression was often accompanied by severe deformation of the dural sac and unclear demarcation from the tumor tissue. In traditional open surgery, it was possible to confirm the boundary of the dural sac and gradually decompress the dural sac by removing part of the adjacent vertebral plate to reveal the normal dural sac. The visual field of the Trans-tubular approach was limited and was more likely to injure the dural sac during the operation. On the other hand, the Trans-tubular approach could not totally remove the posterior structures, and the decompression effect on patients with circumferential compression was not as good as mini-open surgery. Once the tumor recurs, the risk of recompression was higher than that of mini-open surgery, so the patients must receive effective systemic therapy or local radiotherapy after surgery.

It was challenging for patients with local recurrence to identify the tissue structure even with the naked eye due to the hyperplasia of scar tissue. The difficulty would be further increased in the limited field of vision under the tubular. Mini-open surgery had an advantage for patients with recurrent tumors.

There are limitations to the present study:

First, its retrospective and non-randomized nature limited it. However the two groups had no significant difference in baseline data. Selection bias might still affect the results. Second, the relatively small sample size may affect the outcomes available for analysis. A large-scale, prospective, randomized study should be carried out to validate these results. Third, due to missing data of postoperative Hct at 72 h postoperatively, the postoperative Hct in the Gross equation was selected on the first morning after the operation. Patients’ hemodynamics might not be stable at this time, and fluid shifts were not generally complete.

## Conclusions

Decompression through Mini-open or Trans-tubular was safe and effective for patients with spinal metastases. The trans-tubular approach was more suitable for patients with hypo-vascular tumors.

## Data Availability

The data supporting this study’s findings are available on request from the corresponding author. The data are not publicly available due to privacy or ethical restrictions.

## Abbreviations

VAS: Visual Analogue Scale
AIS: American Spinal Injury Association Impairment Scale
ASA: American Society of Anesthesiologists,HGB,haemoglobin
HGB: Haemoglobin
Hct: Haematocrit
Alb: Albumin
